# The Female Perspective of Personality in a Wild Songbird: Repeatable Aggressiveness Relates to Exploration Behaviour

**DOI:** 10.1038/s41598-017-08001-1

**Published:** 2017-08-09

**Authors:** Bert Thys, Rianne Pinxten, Thomas Raap, Gilles De Meester, Hector F. Rivera-Gutierrez, Marcel Eens

**Affiliations:** 1 Department of Biology, Behavioural Ecology and Ecophysiology Group, University of Antwerp, Wilrijk, Belgium; 2 Faculty of Social Sciences, Antwerp School of Education, University of Antwerp, Antwerp, Belgium; 3 Grupo Ecología y Evolución de Vertebrados, Instituto de Biología, Facultad de Ciencias Exactas y Naturales, Universidad de Antioquia, Medellin, Colombia

## Abstract

Males often express traits that improve competitive ability, such as aggressiveness. Females also express such traits but our understanding about why is limited. Intraspecific aggression between females might be used to gain access to reproductive resources but simultaneously incurs costs in terms of energy and time available for reproductive activities, resulting in a trade-off. Although consistent individual differences in female behaviour (i.e. personality) like aggressiveness are likely to influence these reproductive trade-offs, little is known about the consistency of aggressiveness in females. To quantify aggression we presented a female decoy to free-living female great tits (*Parus major*) during the egg-laying period, and assessed whether they were consistent in their response towards this decoy. Moreover, we assessed whether female aggression related to consistent individual differences in exploration behaviour in a novel environment. We found that females consistently differed in aggressiveness, although first-year females were on average more aggressive than older females. Moreover, conform life history theory predictions, ‘fast’ exploring females were more aggressive towards the decoy than ‘slow’ exploring females. Given that personality traits are often heritable, and correlations between behaviours can constrain short term adaptive evolution, our findings highlight the importance of studying female aggression within a multivariate behavioural framework.

## Introduction

In a variety of taxa females often express traits that improve competitive ability (henceforth competitive traits), comparable to the ones expressed in males when they compete for access to mates. Examples of such traits are aggressive behaviour, elaborate ornaments and weaponry^1–3^. However, as females try to maximize their own fecundity, rather than mating *per se*, competitive traits might often target ecological and social resources needed to produce and raise their offspring^3, 4^. In songbirds, females might compete for foraging territories, nest sites and the quality of parental care, and improved access to these resources is generally found to increase female reproductive success^2, 3^. However, competitive trait expression is often costly, since energy and time invested in the expression of competitive traits is no longer available for growth, self-maintenance, or egg production and offspring care^5^.

Female-female aggressiveness is a competitive trait that can serve different, not mutually exclusive functions. During the breeding season, females behaving aggressively towards intruding females might prevent the latter from destroying the eggs and taking over the territory or nest site. Moreover, intruding females might settle nearby with another male thereby reducing food availability and increasing predation risk, or take over the mate of the resident female^6, 7^. Intraspecific aggressiveness between females might hence be used to gain or maintain access to these reproductive resources, but might simultaneously incur costs in terms of an increased risk of injury and decreased energy available for maternal care^2, 5, 7^. Between-individual variation in this type of aggression is hence likely to influence female reproductive fitness and survival^8–10^. Moreover, if females consistently differ in their aggressiveness, this is expected to influence their relative investment in reproduction and survival, i.e. life history trade-offs^5, 11, 12^. However, at present little is known about whether females consistently differ in aggressiveness towards same-sex conspecifics^13–16^.

Consistent, or repeatable, between-individual behavioural differences are commonly referred to as personality^17^. Growing evidence indicates that personality traits are considerably heritable and associated with fitness variation and hence might be both adaptive and generally under selection^8, 18, 19^. Moreover, different aspects of personality are often found to co-vary between individuals forming behavioural syndromes^20^. Given recent evidence that behavioural syndromes can constrain short term adaptive evolution^21^, this highlights the importance of studying behaviour from a multivariate point of view. Hence, if intraspecific aggression between females constitutes a personality trait that is linked within a more general behavioural syndrome, this might have important implications for how females assess life history trade-offs, and hence for the adaptive evolution of consistent individual differences in female aggression^12, 22^.

In a model species, the great tit (*Parus major*), novel environment exploration (henceforth exploration behaviour) has been found to be repeatable and heritable in several populations^23, 24^. So-called ‘fast’ exploring birds typically visit numerous different elements in a novel environment, spending little time exploring each element. In contrast, ‘slow’ exploring birds visit relatively fewer elements but spent more time thoroughly exploring these elements^25^. Exploration behaviour is commonly used as an operational measure of the proactive-reactive personality axis. Proactive individuals, relative to reactive individuals, explore a novel environment faster (i.e. ‘fast’ explorers), readily form routines and are generally thought to be more aggressive (see ref. 26 and references therein). Several studies have looked into the relation between exploration behaviour and different aspects of aggressiveness in male great tits^27–30^. However, female great tits are also known to respond aggressively towards intruding females during the breeding season^6^, but at present it remains to be addressed whether females are consistent in this response, as well as whether female aggression relates with individual differences in exploration behaviour.

Here, we address these questions in a population of free-living great tits. We determined female aggression by assessing the behavioural response of females towards a same-sex decoy (taxidermic mount) during the egg-laying period, and subsequently assessed whether females were consistent in this response. Moreover, we validated whether exploration behaviour was an operational measure of personality in our population, and if so, assessed whether exploration behaviour and aggression constituted a behavioural syndrome in females. In line with findings in male great tits^31^, we expected females to be repeatable in their response towards the decoy. Similarly, exploration behaviour was expected to be repeatable in our population based on previous findings in this species^24^. Furthermore, based on predictions within life history theory^12, 22^ and evidence in males of a variety of species^32^, including great tits^27, 28^, we expected ‘fast’ exploring females to be more aggressive compared to ‘slow’ explorers.

## Material and Methods

### Study population and standard procedures

Data were collected in a suburban nest box population of great tits in the surrounding of Wilrijk, Belgium (51°09′44″N–4°24′15″E). This population has been monitored since 1997 and at present approximately 140 nest boxes are provided for great tits to breed^33–35^. Individuals in the population are provided with a metal leg ring as nestlings (or upon first capture) and all adults are provided with a unique combination of colour rings, allowing identification in the field. Before the onset of the reproductive season, nest boxes were checked regularly for nest building and subsequently they were checked daily to determine the start of egg-laying. Hereafter, the day the first egg was laid is referred to as ‘day 1’. Reproductive activity of all breeding pairs was monitored throughout the breeding cycle as described in Rivera-Gutierrez *et al*.^35^. Age of resident birds was determined using hatching records and immigrant birds were aged (first-year or older) based on plumage characteristics upon first capture^36^.

### Aggression tests

Aggression tests were performed during the breeding season of 2016 (March and April). Each female was subjected to two simulated territorial intrusions during the period of egg-laying, the first on day two and the second on day five. A taxidermic mount of a female great tit (decoy; one out of four) inside a protective wired mesh was placed on top of the focal female’s nest box^6^. After the focal female entered within a 15 m radius around the nest box, or when she was already present at the start of the test, her behaviour was observed for 5 minutes. All tests were performed between 7h30 and 12h00 and observations were made by one of three observers from a distance of 15 m. The observer scored the following aggression parameters: the number of alarm calls produced, the minimum distance to the decoy (approach distance; in meters), the time spent on the decoy (in seconds), and the number of attacks towards the decoy (defined as pecking sequences on the wired mesh directed to the decoy)^6, 31^. Furthermore, we recorded whether or not the female entered the nest box during the test. After each test, the number of eggs in the focal nest box (further referred to as ‘clutch size’) was determined. In order to obtain (repeated) measurements for as many breeding females as possible, tests in which the focal female was not present on day 2 and/or day 5 were repeated the next day (i.e. on day 3 and/or day 6). A total of 321 tests were performed and in 51% of these tests females were present during observations. In this way we obtained 164 measurements on 98 females, representing 93% of the breeding females in the population, with repeated (i.e. two) measurements for 66 of these females. For 51 of the 98 females tested for aggression, exploration scores (see *Exploration behaviour*) were also available. Of these 51 females, 38 were tested once for exploration and 13 were tested twice.

### Exploration behaviour

From 2010 onward, birds in the population (both sexes) were tested for their exploration behaviour as described in Dingemanse *et al*.^23^. In short, individuals roosting in nest boxes in winter (between November and March) were caught, transported to the lab, weighed and measured (tarsus length), and kept overnight in individual cages (0.83 × 0.4 × 0.5 m; 16 cages in total) connected to an exploration room (4.0 × 2.4 × 2.3 m) containing five artificial trees. On the morning following capture, individual birds were released into the exploration room via a sliding door, which directly connects the individual cage with the room (see ref. 37 for details). Exploration scores were calculated as the total number of hops and flights within the first 2 minutes upon arrival in the room^23^. After the test, individuals were released at the outdoor location where they had been captured. Following this procedure, a total of 685 exploration test on 550 individuals (N_females_ = 250; N_males_ = 300) were performed in the winters of 2010 until and including 2015, with one (N = 427), two (N = 113), three (N = 9) or four (N = 1) tests per individual.

### Statistical analyses

All analyses were performed in R 3.3.1 (R Core Team, 2014). The sim function (package arm^38^) was used throughout to simulate values of the posterior distribution of all model parameters. Results are presented as estimated means with 95% credible intervals (CrI), unless stated otherwise, and effects were considered ‘significant’ in the frequentist sense when CrI’s did not overlap zero.

#### Principal component analysis

The parameters scored during the aggression tests are inherently linked by their nature. That is, females that spend time on the decoy will have a closer approach distance, and spending time on the decoy is a prerequisite for the occurrence of attacks. Moreover, the display of some aggression parameters is mutually exclusive. Females are never observed to produce alarm calls while sitting on the decoy, as alarm calls are always being produced only from a distance (B.﻿T.,﻿ pers. obs.). Similarly, while females enter the nest box they cannot simultaneously display the other behaviours. In an attempt to capture the correlation structure between aggression parameters, we performed a principal component analysis (PCA). Aggression parameters were standardized prior to analysis and principal components (PCs) with eigenvalues >1 were used in further analyses. Parameters with factor loadings >0.3 were considered to contribute importantly to a component^39–41^. Approach distance to the decoy was multiplied by −1 prior to analysis so that higher values represented more aggressive responses^31^.

#### Univariate mixed models

To model variation in the aggression PCs we fitted univariate mixed models (lmer function, package lme4^42^) with Gaussian error distribution and random intercepts for female identity (Female ID; N = 98). Random intercepts for observer (N = 3) and decoy (N = 4) were included to control for variation due to specific characteristics of the decoy and observer. Each model included the following fixed effects as standardized continuous covariates: ‘clutch size’ (number of eggs at the moment of the test), ‘Julian date’ (days since July first, log-transformed) and ‘start time’ of the test (expressed as minutes after sunrise). Female ‘age class’ was included as a three-level factor (i.e. first-year, older, or unknown age).

Exploration scores (both sexes) were square-root transformed (to meet model assumptions), and standardized. Variation in exploration scores was modelled using a univariate mixed model with Gaussian error distribution and random intercept for bird identity (ID; N = 550). To control for year-specific variation in exploration scores we also included a random intercept for ‘year’ (2010–2015). Here, ‘year’ refers to the winter season of a respective year (i.e. all test performed during a given winter season are indicated by the same year). Based on findings in Dingemanse *et al*.^24^, we included the following fixed effects: ‘sequence’ as a two-level categorical factor (first versus repeated test; with repeated test including second, third and fourth test), ‘interval’ (days since the previous test), and ‘Julian date’ (days since July first, log-transformed). Julian date was centered within individuals to separate within- from between-individual effects^43^ and both components were added as fixed effects in the model. This allowed to test for the possibility that certain types of individuals are more likely to be caught at certain times of the year (between-individual effect; i.e. mean date per individual) and whether individuals changed their exploration behaviour over the year (within-individual effect; i.e. for all observations of an individual the deviation from its mean date; cf. ref. 24). Furthermore, ‘sex’ and an index for body condition (residuals from an ordinary least square regression of body mass on tarsus length^44^) were included as fixed effects. Moreover, since sexes might differ in the repeatability of behaviours (reviewed in ref. 45), we performed similar univariate mixed models for the sexes separately.

Individuals with single measurements were included in all univariate models, given that they contribute in estimating between-individual variances^46^. Stepwise backward elimination of non-significant terms, starting with the least significant, was used to obtain minimum adequate models (MAMs). Adjusted repeatabilities were calculated from these MAMs as the between-individual variance divided by the sum of the between-individual and residual variance^46^.

To test whether variation in aggression PCs related to variation in exploration scores, we fitted a second set of univariate mixed models using the subset of females for which both aggression and exploration scores were available (N = 51). To this end, we extracted best linear unbiased predictors (BLUPs) of the random effect (i.e. Female ID) from the MAM of exploration scores (female data only). Best linear unbiased prediction is a method to obtain point estimates of a random effect in a mixed model^47^. In this case, the BLUPs represent the individual-specific values for exploration scores after controlling for confounding factors included in the model (see e.g. ref. 40). Subsequently, these exploration BLUPs were included as a fixed effect in the univariate mixed models with aggression PCs as response variable. These models were fitted with a Gaussian error distribution and random intercepts for Female ID, and included the fixed effects which were found in the previous analysis on the overall dataset to influence aggression PCs. For each model we calculated the conditional coefficients of determination (R²_GLMM(c)_) and the total variance in aggression PCs explained by exploration BLUPs (r²; see refs 48–50). To provide additional information on how each separate aggression parameter related to exploration behaviour, we performed similar univariate mixed model analyses for aggression parameters separately (see Supplementary Information).

### Ethical statement

This study was approved by the ethical committee of the University of Antwerp (ID numbers 2011–31 and 2014–45) and performed in accordance with Belgian and Flemish laws. The Belgian Royal Institute for Natural Sciences (Koninklijk Belgisch Instituut voor Natuurwetenschappen) provided ringing licences for authors and technical personnel.

## Results

### Aggression parameters and PCA

Descriptive statistics of separate aggression parameters are given in Supplementary Table S1. The PCA of the aggression parameters produced two principal components with eigenvalues >1, together explaining 65% of the total variance (Table 1). Higher scores on the first component (PC1; explaining 43% of the variance) reflected closer approach distance, more time on the decoy, more attacks, and a higher likelihood to enter the nest box. On the other hand, low scores on PC1 reflected more alarm calls produced at larger distances from the decoy. The second component (PC2) explained 22% of the variance and reflected a contrast between entering the nest box (low scores) and the other aggression parameters (high scores).

**Table 1:** PCA loadings for aggression parameters scored during simulated territorial intrusion in female great tits (N = 98).

	PC1	PC2
Eigen value	1.46	1.06
Proportion total variance	0.43	0.22
No. call**s**	**−0.327**	0.191
Time on decoy	**0.569**	**0.390**
No. attacks	**0.530**	**0.458**
Approach distance	**0.399**	**−0.415**
Enter nest box (Y/N)	**0.361**	**−0.656**

Approach distance was multiplied by −1 prior to analysis. Parameters contributing importantly to a component are highlighted in bold.

### Sources of variation in aggression PCs

**Figure 1:**
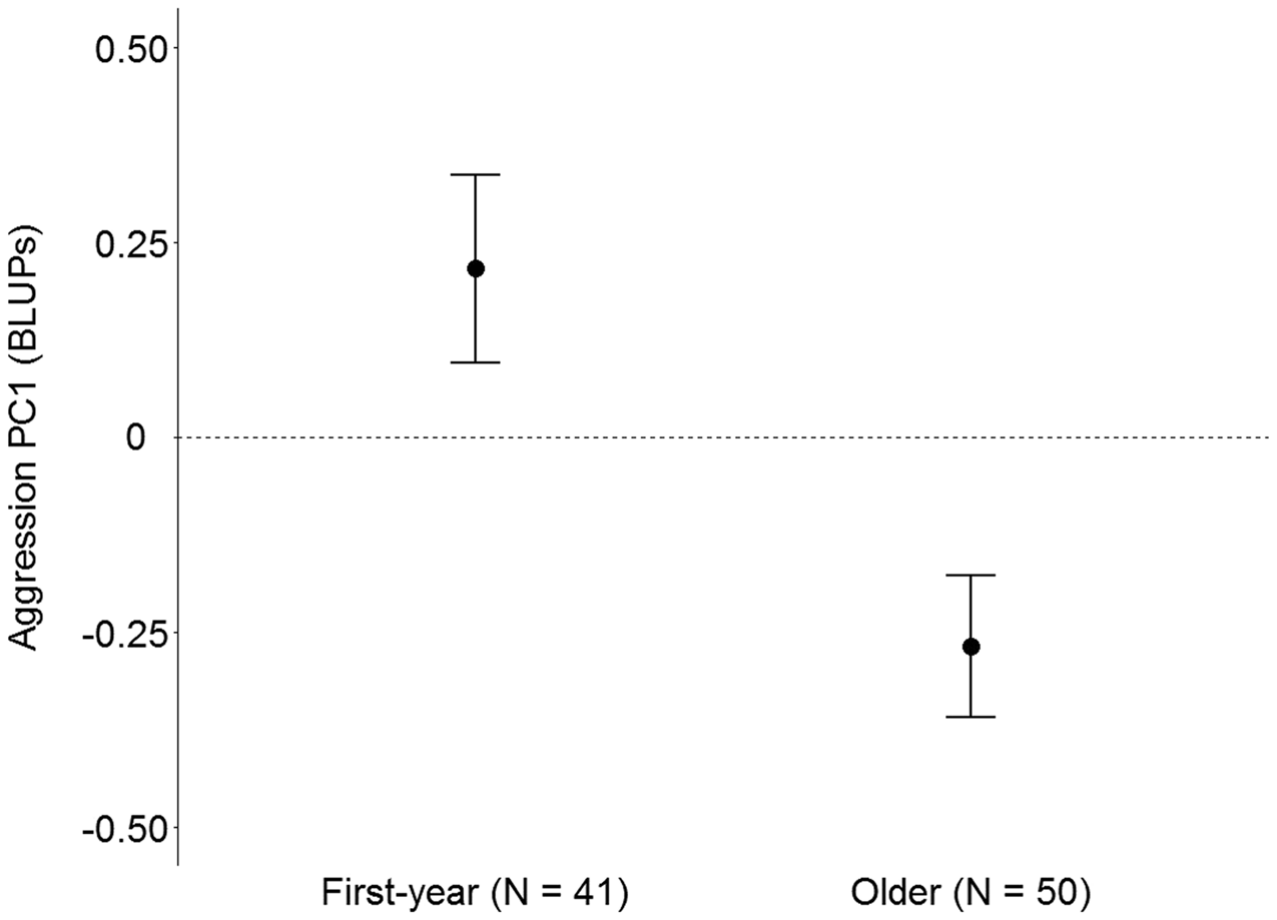
First-year females are more aggressive than older females, effect of age class on averageaggression PC1 scores. Points indicate mean aggression PC1 scores (best linear unbiased predictors(BLUPs) from model with random intercepts for individual) and error bars indicate standard errors. Higher values indicate higher aggressiveness, and lower values indicate lower aggressiveness.

Between-individual differences explained a substantial part of the phenotypic variation in the aggression PCs (Table 2). The adjusted repeatability for PC1 was 0.43 [0.33; 0.53], while PC2 had a repeatability of 0.21 [0.15; 0.29]. Since CrI’s for repeatabilities are far from overlapping zero, these results provide strong support for the presence of between-individual variation in female aggression. Age class influenced PC1 scores, with first-year females on average having higher scores compared to older females (Fig. 1). None of the other variables (clutch size, Julian date and start time) had an effect on PC1. There was also no support for an effect of any of the variables, including age class, on PC2 (Table 2). The identity of the observer or decoy explained little to no variation, except for PC1 where observer differences were small but present (Table 2). Separate aggression parameters were also found to be repeatable (all R > 0.52; see Supplementary Table S2).

**Table 2:** Sources of variation in aggression principal components for female great tits (N = 98).

	Aggression PC1	Aggression PC2
**Fixed effects**	β (95% CrI)	β (95% CrI)
Intercept	0.30 (−0.29; 0.88)	0.00 (−0.18; 0.17)
Clutch size^a^	0.01 (−0.19; 0.19)	0.10 (−0.07; 0.27)
Julian date	−0.14 (−0.37; 0.10)	−0.17 (−0.34; 0.01)
Start time	0.12 (−0.08; 0.33)	0.09 (−0.07; 0.26)
Age 2^b^	**−0.81 (−0.31; −1.30)**	0.00 (−0.37; 0.34)
Age 3^b^	0.71 (−0.21; 1.162)	0.01 (−0.67; 0.79)
**Random effects**	σ² (95% CrI)	σ² (95% CrI)
Female ID	0.79 (0.60; 1.01)	0.24 (0.17; 0.32)
Observer	0.10 (0.01; 0.28)	0.00 (0.00; 0.00)
Decoy	0.01 (0.00; 0.04)	0.00 (0.00; 0.00)
Residual	1.05 (0.85; 1.33)	0.89 (0.72; 0.11)
**Repeatability**	r (95% CrI)	r (95% CrI)
	0.43^#^ (0.33; 0.53)	0.21^$^ (0.15; 0.29)

Point estimates for fixed (β) and random (σ²) parameters, as well as adjusted repeatabilities (r), are given with 95% credible intervals (CrI). Fixed effects where CrI’s do not overlap zero are highlighted in bold. aNumber of eggs in the clutch at the moment of aggression testing. ^b^Age class: ‘first-year’ (N = 41) is used as reference category, ‘Age 2’ is older (N = 50) and ‘Age 3’ is unknown age (N = 7). ^#^Calculated based on minimum adequate model (MAM) including Age class as fixed effect and random intercepts for Female ID and Observer (R²_GLMM(c)_ = 0.52). ^$^Calculated based on MAM including random intercepts for Female ID (R²_GLMM(c)_ = 0.21).

### Sources of variation in exploration scores

Exploration scores ranged between 0 and 53 (mean ± s.e.m.: 11.2 ± 10.0). Overall, the adjusted repeatability of exploration scores was 0.40 [0.36; 0.45], strongly supporting the presence of substantial differences between individuals (Supplementary Table S3). Exploration scores changed with sequence, with an increase from the first to subsequent tests. The effects of interval and Julian date were not supported in our population and also body condition did not influence exploration scores. Year did explain a part of the phenotypic variation in exploration scores (Supplementary Table 3).

However, although exploration scores did not differ on average between the sexes (Supplementary Table S3), females tended to be less repeatable (R = 0.34 [0.29–0.41]; Table 3) than males (R = 0.47 [0.41–0.53]; Supplementary Table S3). This sex difference in exploration score repeatability was caused by greater within-individual variance in females (0.62 [0.53; 0.72]) than males (0.44 [0.38; 0.51]), as indicated by non-overlapping credible intervals, not by sex differences in between-individual variances (females: 0.32 [0.27; 0.39]; males: 0.39 [0.33; 0.45]). Given this finding we used in further analyses only the output (i.e. BLUPs) of the model for female exploration scores.

**Table 3:** Sources of variation in female exploration scores (ES) from a free-living great tit population in the surroundings of Wilrijk, Belgium (N = 250).

	**Female ES**
***Fixed effects***	β (95% CrI)
Intercept	−0.05 (−0.30; 0.20)
Sequence^a^	**0**.**45** (**0**.**15; 0**.**76**)
Interval	−0.07 (−0.27; 0.14)
Julian date av^b^	0.11 (−0.01; 0.23)
Julian date dev^b^	0.02 (−0.09; 0.12)
Body condition	−0.10 (−0.25; 0.05)
***Random effects***	σ² (95% CrI)
ID	0.32 (0.27; 0.39)
Year^c^	0.07 (0.02; 0.15)
Residual	0.62 (0.53; 0.72)
**Repeatability**	r (95% CrI)
	0.34^#^ (0.29; 0.41)

Point estimates for fixed (β) and random (σ²) parameters, as well as adjusted repeatabilities (r), are given with 95% credible intervals (CrI). Fixed effects where CrI’s do not overlap zero are highlighted in bold. Results of similar analyses for ES data of the sexes combined and male data only can be found in Supplementary Table S3. ^a^‘first test’ is used as reference category. ^b^Represent between- (av) and within- (dev) individual component of the Julian date, after within-individual centering. ^c^Winter seasons from 2010 to 2015. ^#^Calculated based on minimum adequate model (MAM) including Sequence as fixed effect and random intercepts for ID and Year (R²_GLMM(c)_ = 0.40).

### Relation between exploration and aggressiveness

**Figure 2:**
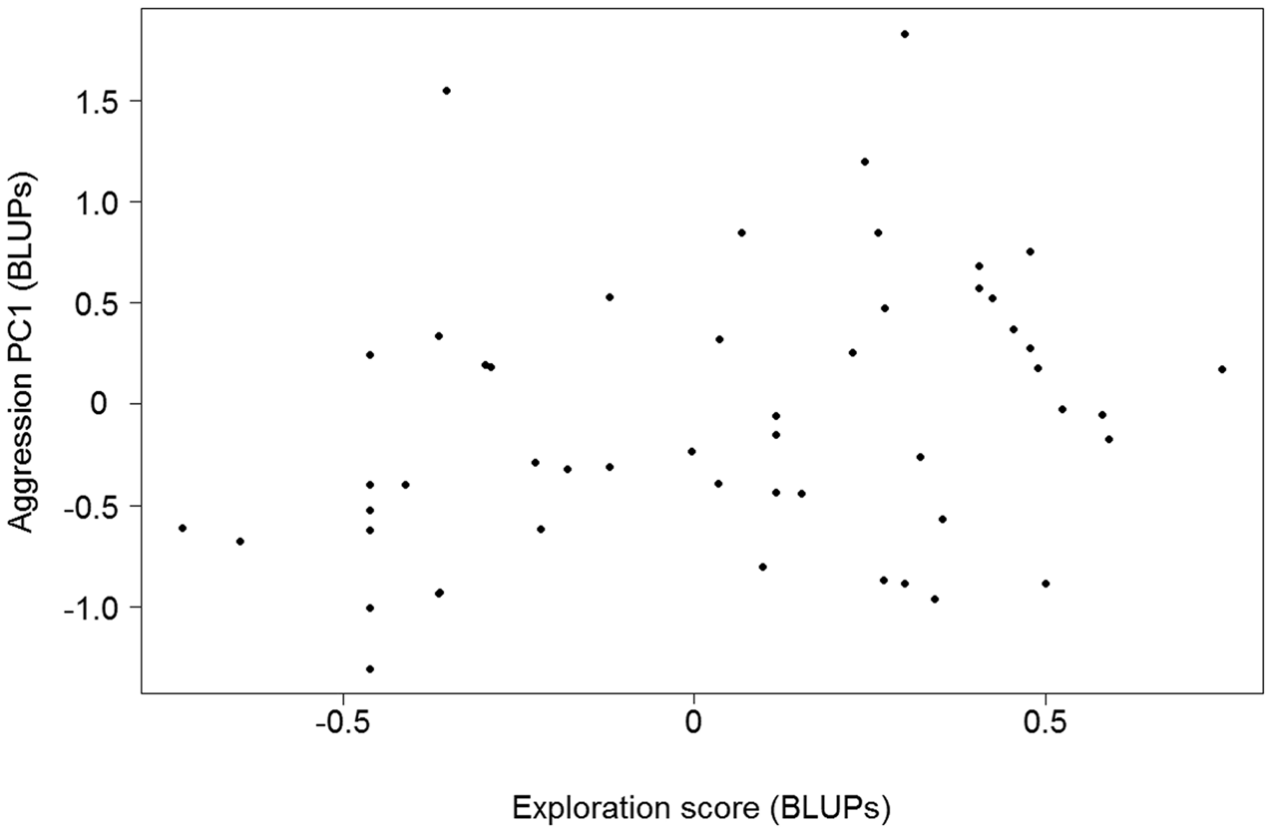
Plot of the relationship between aggressiveness (PC1) and exploration scores in female great tits (N = 51) on the individual level. To illustrate the individual-level relationship we plotted best linear unbiased predictors (BLUPs) from the minimum adequate model (MAM) for aggression PC1 (see Table 2) against the BLUPs of the MAM for female exploration scores (see Table 3). Real analysis was based on a univariate mixed model (see Statistical analyses) and more exploratory females were found to be more aggressive (regression coefficient β = 0.87 [0.08, 1.63]).

Female aggression PC1 related ‘significantly’ and positively with female exploration scores (BLUPs; regression coefficient β = 0.96 [0.14, 1.89]; r² = 0.11; see also Fig. 2). On the other hand, we found no support for a relationship between aggression PC2 and exploration scores (BLUPs; regression coefficient β = −0.21 [−0.86, 0.40]; r² = 0.01). For separate aggression parameters, we found that the number of alarm calls related ‘significantly’ and negatively with female exploration scores (regression coefficient β = −1.98 [−3.70, −0.34]; r² = 0.10) while time on the decoy showed a positive relationship (regression coefficient β = 3.84 [0.52, 7.03]; r² = 0.13). We found no support for a relationship between the other aggression parameters (occurrence of attack, approach distance, enter nest box) and exploration scores (see Supplementary Table S4).

## Discussion

Our study reveals that free-living female great tits show consistent individual variation in aggressive behaviour towards a female decoy during the egg-laying period. Aggressiveness in females ranged from individuals that spend relatively more time on the decoy, attacking, and likely entering their nest box (more aggressive), to individuals that produced relatively more alarm calls from a larger distance (less aggressive). First-year females were on average more aggressive than older females. Exploration behaviour was also found to be a repeatable personality trait in our population, although females tended to be less consistent in their exploration behaviour than males. Nevertheless, we found that individual variation in female aggressiveness related to exploration behaviour. Specifically, conform predictions within life history theory, ‘fast’ exploring females were more aggressive during the territorial intrusion than ‘slow’ exploring females. Taken together our results indicate that free-living female great tits show consistent between-individual differences in aggressiveness which relate to a commonly used operational measure of personality in this species. In what follows we discuss the potential implications of our findings for the reproductive trade-offs that females might experience, and hence the adaptive evolution of consistent individual differences in female aggression.

### Consistency in female-female aggressiveness

Variation in aggressiveness among females was mainly described by a single behavioural axis. This axis reflected whether individuals were more likely to physically approach the decoy (spending time on the decoy, attacking, and entering the nest box) or whether they were more likely to respond vocally (by producing alarm calls) from a relatively larger distance. Females were moderately repeatable in their response (R = 0.43), comparable to the average repeatability of behaviour^51^. A smaller amount of variation reflected whether females entered their nest box or spent their time outside, engaging in other aggressive behaviours (i.e. spending time on the decoy and attacking). However, females were significantly less consistent in this latter component (R = 0.21), as indicated by non-overlapping credible intervals of the repeatability estimates for both principal components. Also separate aggression parameters were found to be highly repeatable, which provides further support for the presence of substantial differences in aggressiveness among females. The few studies that addressed the consistency of same-sex aggression in females either used captive tests on individuals temporarily taken from the wild^14, 15^, or studied free-living animals but assessed aggression only repeatedly for few individuals^13^. Hence, validation of our findings requires more field-based studies that repeatedly assess aggression in large numbers of free-living females, across a variety of species.

The here observed consistent individual differences in aggression among females are expected to influence female reproductive success and survival (reviewed by ref. 8). However, the costs and benefits associated with aggression in females might be complex^5, 52, 53^, and at present our understanding of how consistent individual differences in aggression influence these costs and benefits is limited. Physically approaching and attacking an intruding female conspecific might be an effective means of deterring her from the territory, preventing her from destroying the eggs or taking over the nest site and/or mate^6^. However, consistently behaving aggressively might result in an increased risk of injury, and a reduction in energy available for egg production and offspring care^2, 54^. Simultaneously, higher levels of aggression are often associated with higher levels of androgens^53, 55^, which are expected to negatively influence female fertility^56^. Individual differences in aggression might also influence survival if they are maintained across different ecological situations, such as for example in response towards predators^16, 57^. Consequently, individuals with more aggressive phenotypes might outcompete conspecifics for limited reproductive resources, but behave unsuitably aggressive towards predators, exposing them to greater risks^20^. Hence, better insight into the costs and benefits of female aggression requires a more solid understanding of individual differences in female aggression across ecologically relevant situations, including its proximate underpinning. Moreover, long term data on associated fitness consequences and its link with other behaviours are needed to determine the adaptive significance of individual differences in female aggression^58^.

Females consistently differed in their aggressive behaviour, but we found that first-year females were on average more aggressive than older females. Although similar population-level age effects have been found in males of some passerine species (see ref. 59 and references therein), we have no clear explanation for our observation. Uncertainty about obtaining and maintaining a territory might be higher for first-year than older females. Indeed, breeding area fidelity is very high in great tits and adults normally reoccupy their territory from the previous year^60, 61^. Consequently, first-year females might have to invest more in aggressively defending their recently established territory compared to older females. This seemingly contrasts predictions within life history and asset protection theory, since the investment in reproduction and associated risk-taking behaviour is expected to increase with age due to a decline in future fitness expectations (i.e. assets)^11, 12^. In other words, younger females are expected to have high future fitness expectations (i.e. more assets to protect) and should behave less aggressive in order to survive and harvest these assets, while older females, having lower future fitness expectations, should behave more aggressive to favour current reproduction^12, 22^. However, if aggressiveness is indeed under survival selection as predicted within life history theory^8, 22^, more aggressive first-year females might simply not survive until the next breeding season and hence selectively disappear from the population, resulting in on average less aggressive phenotypes in older age classes^62^. Moreover, since age differences at the population level do not necessarily reflect changes at the within-individual level^62^, another possible explanation is that females become less aggressive with age due to senescence; i.e. a within-individual decline in aggressiveness associated with lower reproductive success^63, 64^. Longitudinal data on aggression of the same individuals across ages is necessary to explore whether the here observed difference between age classes is the result of selective disappearance of aggressive first-year females as predicted by life history theory, senescence of aggression within individuals, or both^62, 64^.

### Exploration behaviour and female aggressiveness

Exploration behaviour in a novel environment was moderately repeatable in our population of great tits, with a repeatability estimate highly comparable to studies in other West-European populations (R = 0.40)^24^. This implies that the commonly used novel environment test also captures consistent behavioural differences between individuals in our population of great tits. Exploration behaviour increased from the first to subsequent tests (independent of interval between tests or seasonal effects), potentially because of habituation to novelty with repeated testing, in line with findings in other great tit populations^24^.

Although sexes did not differ on average in their exploration behaviour, females appeared to be less consistent than males as a consequence of greater behavioural plasticity (i.e. within-individual variation) in the former. Sex differences in behavioural consistency are not uncommon^41, 45, 65^, but our findings contrasts those of an earlier study in great tits that showed no sex differences in the repeatability of exploration behaviour^23^. On the other hand and in line with our findings, captive zebra finch females (*Taeniopygia guttata*) were not less exploratory on average but were less consistent in their novel environment exploration than males^65^. Given that the here observed sex difference in repeatability was caused by higher within-individual variation in females than males (and not because of sex differences in between-individual variation), one might argue that selection is acting to a greater extent on the degree of consistency in males than in females. However, the exact nature of selection pressures responsible for our observation, whether sexual (i.e. *via* mate choice and/or intrasexual competition) or natural (e.g. due to sex differences in ecological demands), remains to be determined (for full discussion, see ref. 45).

Nevertheless, we found that female exploration behaviour related to consistent individual differences in female aggression (PC1). When we analysed each individual aggression parameter in relation to exploration behaviour we found that the number of alarm calls and the time spend on the decoy related ‘significantly’ to exploration behaviour. Despite that not all individual aggression parameters relate to exploration behaviour, there are several interlinked reasons to believe that the aggression PC1 adequately reflects differences in aggressiveness, which moreover relates to individual differences in exploration behaviour. First of all, although ‘not significant’, the relationships of the other three aggression parameters (occurrence of attack, approach distance and enter nest box) with exploration behaviour are in the same direction as how these parameters correlate to PC1 (Table 1; Supplementary Table S4). Secondly, since attacks can only occur when females spend time on the decoy, the latter is likely to reflect the intensity of female aggressiveness (see also refs 6 and 66), and here we show that this measure relates positively with exploration behaviour. Lastly, the observed negative relationship between the number of alarm calls and exploration behaviour is in further support of this, since females are never observed to produce alarm calls while sitting on the decoy. Hence, aggressiveness and exploration behaviour form a behavioural syndrome, with ‘fast’ exploring females being more aggressive (more time on the decoy, potentially attacking) during territorial intrusion than ‘slow’ exploring females (producing more alarm calls at a distance from the decoy).

Such an aggression-exploration syndrome has been found in a variety of species^32^, but most previous studies have focused on aggressiveness in males. Hence we provide scarce evidence that female aggression is a personality trait that is part of a more general behavioural syndrome with exploration behaviour in a small passerine, the great tit. Given that personality traits are often heritable^19, 23^, and that behavioural syndromes might constrain short term adaptive evolution^21^, our findings highlight the importance of studying female aggressiveness within a multivariate behavioural framework.

The observed relationship between exploration behaviour and female aggression is in line with predictions within life history theory, which suggests that personality differences are the result of trade-offs in life history traits^12, 22^. As stated earlier, this theory predicts that females with low future fitness expectations (‘assets’) behave consistently more aggressively and engage in more risky behaviours such as exploration, in order to maximize their current reproductive fitness (at the cost of survival), whereas less aggressive, ‘slow’ exploring females do the opposite^12, 22^. Along this line, several studies in both male and female great tits have shown that exploration behaviour relates to risky behaviours in a variety of ecological contexts^37, 67–70^. Most notably, a recent experimental study revealed that ‘slow’ exploring females, relative to ‘fast’ explorers, took less risk in response to novelty during incubation^70^. Together, this suggests that less aggressive, ‘slow’ exploring females might prioritize survival over reproductive investment, and hence differ in life history strategy compared to more aggressive, ‘fast’ exploring females, who might prioritize reproduction^12^. Moreover, if more aggressive, ‘fast’ exploring females indeed prioritize reproduction over survival, then selective disappearance of more aggressive phenotypes with age might explain the observed population-level age effect, but this requires further investigation (see also above). Hence, whether life history trade-offs favour the adaptive evolution of the here observed individual variation in female aggressiveness remains to be determined, and requires long term data on aggressiveness of the same individuals across years and data on life time fitness variation^58^.

## Conclusion

Here we have provided field-based empirical evidence that female great tits show consistent between-individual differences in aggressiveness which relate to a commonly used operational measure of personality in this species. Our findings highlight that a more solid understanding of the influence of aggressiveness on reproductive trade-offs requires the integration of female aggression within a multivariate behavioural framework. Specifically, future research should aim to investigate how consistent individual differences in female aggression influence both the access to resources and investment in maternal care^52, 53, 71^. Simultaneously, determining whether consistent differences in female aggression evolved as a consequence of trade-offs between current and future reproduction requires insights into its link with other personality traits, and long term data on associated fitness consequences.

## Electronic supplementary material

Supplementary information
