# Supplementary material for: The Female Perspective of Personality in a Wild Songbird: Repeatable Aggressiveness Relates to Exploration Behaviour
Source: Sci Rep. 2017 Aug 9;7:7656. doi: 10.1038/s41598-017-08001-1 (PMC5550452; doi:10.1038/s41598-017-08001-1)
Supplement: Supplementary file 1 — Supplementary information [file 41598_2017_8001_MOESM1_ESM.pdf]

Supplementary information

The female perspective of personality variation in a wild songbird: repeatable aggressiveness relates to exploration behaviour

Bert Thys[1,*], Rianne Pinxten[1,2], Thomas Raap[1], Gilles de Meester[1], Hector F. Rivera-Gutierrez[1,3], Marcel Eens[1]

[1]Department of Biology, Behavioural Ecology and Ecophysiology Group, University of Antwerp, Wilrijk, Belgium

[2]Faculty of Social Sciences, Antwerp School of Education, University of Antwerp, Antwerp, Belgium

[3]Grupo Ecología y Evolución de Vertebrados, Instituto de Biología, Facultad de Ciencias Exactas y Naturales, Universidad de Antioquia, Medellín, Colombia

[*]Corresponding author:

Bert Thys

Campus Drie Eiken, Building D - Room 1.23

Universiteitsplein 1, 2610 Antwerp (Wilrijk), Belgium

Email: bert.thys@uantwerpen.be

Contents:

Supplementary Table S1: descriptive statistics of separate aggression parameters

Supplementary Methods: statistical analyses of separate aggression parameters

Supplementary Table S2: sources of variation in separate aggression parameters

Supplementary Table S3: sources of variation in exploration scores

Supplementary Table S4: relationship between exploration and aggression parameters

Supplementary References

Supplementary Table S1: Descriptive statistics of the aggression parameters scored during simulated territorial intrusion in female great tits (number of tests = 164; duration: 5 minutes). Mean, standard error (s.e.m.), minimum (min), median and maximum (max) values are given, and % refers to the percentage of aggression tests in which females were observed to perform the respective aggression parameter. For the parameter 'Time on decoy' % refers to the percentage of tests in which females spent at least one second on the decoy.

Parameter	mean	s.e.m.	min	median	max	%
No. calls	18.8	2.4	0	5.5	196	65.2
Time on decoy (s)	37.7	4.6	0	5	241	56.1
No. attacks	3.3	0.7	0	0	63	28.7
Approach distance (m)	1.6	0.2	0	0	14	NA
Enter nest box	NA	NA	NA	NA	NA	40.2

Supplementary Methods: statistical analyses of separate aggression parameters

Sources of variation in aggression parameters

Variation in each of the aggression parameters was modelled separately using the MCMCglmm package[1] in R (R core team, 2014), which relies on Markov Chain Monte Carlo sampling to estimate parameters. Relatively uninformative priors were used throughout and convergence and mixing of models was assessed by visually checking traces of posterior distributions over iterations and Gelman-Rubin statistics between chains[2]. Each model included random intercepts for Female ID (N = 98), and the following fixed effects as standardized continuous covariates: 'clutch size' (number of eggs at the moment of the test), 'Julian date' (days since July first, log-transformed) and 'start time' of the test (expressed as minutes after sunrise). Female 'age class' was included as a three-level factor (i.e. first-year, older, or unknown age). The different aggression parameters were modelled with the following error distribution: approach distance (square-root transformed) with Gaussian errors, the number of

calls and time on the decoy (untransformed) with Poisson-errors, and whether females entered the nest box (yes/no) and attacked (occurrence of attack; yes/no) with binomial errors. Adjusted repeatabilities were calculated as the between-individual variance divided by the sum of the between-individual and residual variance[3]. Conditional coefficients of determination ($R^2_{GLMM(c)}$) were calculated following methods described in Nakagawa & Schielzeth[4]. Results are presented in Supplementary Table S2.

Relationship between exploration and aggression parameters

The relationship between each aggression parameter and exploration (BLUPs) was assessed for the subset of females for which both aggression and exploration scores were available (N = 51). Univariate mixed models were fitted as described above, but included only the fixed effects found to influence the respective aggression parameter in the total dataset (see Supplementary Table S2), and included female exploration BLUPs as continuous fixed effect. Conditional coefficients of determination ($R^2_{GLMM(c)}$) and the total variance in aggression parameters explained by exploration BLUPs (r^2; see[5]) were calculated following methods described in Nakagawa & Schielzeth[4] and Ray-Mukherjee et al[6]. Results are presented in Supplementary Table S4.

Supplementary Table S2: Sources of variation in aggression parameters for female great tits (N = 98). Point estimates for fixed (β) and random (σ^2) parameters, as well as adjusted repeatabilities (r), are given with 95 % credible intervals (CrI). Fixed effects where CrI's do not overlap zero are highlighted in bold.

	No. calls	Time on decoy	Occurrence of attack	Approach distance	Enter nest box
**Fixed**	β (95% CrI)	β (95% CrI)	β (95% CrI)	β (95% CrI)	β (95% CrI)
Intercept	0.60<br>(-0.24 ; 1.41)	1.50<br>(0.26 ; 2.51)	-1.22<br>(-2.84 ; 0.17)	0.58<br>(0.28 ; 0.89)	0.04<br>(-1.05 ; 1.03)
Clutch size[a]	**0.44**<br>**(0.10 ; 0.79)**	-0.32<br>(-0.92 ; 0.22)	-0.09<br>(-0.73 ; 0.69)	-0.02<br>(-0.17 ; 0.13)	-0.19<br>(-0.77 ; 0.35)
Julian date	-0.56<br>(-1.06 ; 0.02)	-0.03<br>(-0.77 ; 0.80)	-0.24<br>(-1.51 ; 0.68)	0.07<br>(-0.16 ; 0.26)	0.54<br>(-0.17; 1.36)
Start time	0.00<br>(-0.38 ; 0.40)	0.19<br>(-0.40 ; 0.78)	0.49<br>(-0.31 ; 1.34)	0.00<br>(-0.17 ; 0.15)	-0.13<br>(-0.66 ; 0.50)
Age 2[b]	0.84<br>(-0.16 ; 1.86)	**-1.81**<br>**(-3.43 ; -0.38)**	**-2.06**<br>**(-4.43 ; -0.13)**	0.26<br>(-0.17 ; 0.66)	**-1.68**<br>**(-3.30 ; -0.17)**
Age 3[b]	-0.27<br>(-2.52 ; 1.85)	0.84<br>(-1.97 ; 3.70)	1.60<br>(-2.02 ; 5.68)	-0.28<br>(-1.01 ; 0.54)	1.77<br>(-1.09 ; 4.78)
**Random**	σ^2 (95% CrI)	σ^2 (95% CrI)	σ^2 (95% CrI)	σ^2 (95% CrI)	σ^2 (95% CrI)
Female ID	3.92<br>(1.96 ; 6.53)	7.88<br>(3.11 ; 12.67)	11.02<br>(0.64 ; 28.68)	0.57<br>(0.27 ; 0.87)	5.78<br>(0.18 ; 14.28)
Residual	2.57<br>(1.43 ; 4.08)	5.97<br>(2.92 ; 8.93)	1.00<br>(1.00 ; 1.00)	0.56<br>(0.38 ; 0.77)	1.00<br>(1.00 ; 1.00)
Repeatability	r (95% CrI)	r (95% CrI)	r (95% CrI)	r (95% CrI)	r (95% CrI)
	0.59[#]<br>(0.37 ; 0.73)	0.56[*]<br>(0.32 ; 0.75)	0.72[$]<br>(0.30 ; 0.92)	0.52[µ]<br>(0.30 ; 0.68)	0.59[£]<br>(0.16 ; 0.83)

[a] number of eggs in the clutch at the moment of aggression testing

[b] Age class: 'first-year' (N = 41) is used as reference category, 'Age 2' is older (N = 50) and 'Age 3' is unknown age (N = 7)

[#] calculated based on minimum adequate model (MAM) including Day as fixed effect and random intercepts for Female ID ($R^2_{GLMM(c)}$ = 0.96)

[*]calculated based on MAM including Age class as fixed effect and random intercepts for Female ID ($R^2_{GLMM(c)}$ = 0.98)

[$] calculated based on MAM including Age class as fixed effect and random intercepts for Female ID ($R^2_{GLMM(c)}$ = 0.41)

[µ] calculated based on MAM including random intercepts for Female ID ($R^2_{GLMM(c)}$ = 0.53)

[£] calculated based on MAM including Age class as fixed effect and random intercepts for Female ID ($R^2_{GLMM(c)}$ = 0.34)

Supplementary Table S3: Sources of variation in exploration scores (ES) for both sexes combined (N = 550) and males only (N = 300). Point estimates for fixed (β) and random (σ^2) parameters, as well as adjusted repeatabilities (r), are given with 95 % credible intervals (CrI). Fixed effects where CrI's do not overlap zero are highlighted in bold. Results of a similar analysis for female data only can be found in Table 3 in the main text.

	**ES Both sexes**	**ES Males**
Fixed effects	β (95% CrI)	β (95% CrI)
Intercept	0.01 (-0.34 ; 0.40)	-0.10 (-0.45 ; 0.26)
Sex[a]	0.05 (-0.10 ; 0.20)	-
Sequence[b]	**0.39 (0.22 ; 0.57)**	**0.34 (0.13 ; 0.57)**
Interval	0.09 (-0.04 ; 0.24)	0.01 (-0.11 ; 0.14)
Julian date av[c]	0.00 (-0.08 ; 0.08)	-0.10 (-0.20 ; 0.01)
Julian date dev[c]	-0.03 (-0.10 ; 0.04)	-0.05 (-0.12 ; 0.02)
Body condition	-0.07 (-0.14 ; 0.02)	-0.06 (-0.19 ; 0.06)
Random effects	σ^2 (95% CrI)	σ^2 (95% CrI)
ID	0.35 (0.31 ; 0.39)	0.39 (0.33 ; 0.45)
Year[d]	0.22 (0.06 ; 0.49)	0.19 (0.05 ; 0.41)
Residual	0.51 (0.46 ; 0.57)	0.44 (0.38 ; 0.51)
Repeatability	r (95% CrI)	r (95% CrI)
	0.40[#] (0.36 ; 0.45)	0.47[#] (0.41 - 0.53)

[a] 'female' is used as reference category
[b] 'first test' is used as reference category
[c] represent between- (av) and within- (dev) individual component of the Julian date, after within-individual centering
[d] winter seasons from 2010 to 2015
[#] calculated based on minimum adequate model (MAM) including Sequence as fixed effect and random intercepts for ID and Year. Both sexes: $R^2_{GLMM(c)}$ = 0.67; males only: $R^2_{GLMM(c)}$ = 0.58

Supplementary Table S4: Results of univariate mixed models used to model the relationship between separate aggression parameters and female exploration scores [ES (BLUPs); N = 51]. Fixed effects found to influence aggression parameters in the overall dataset were included in these models (see Supplementary Table S2). Point estimates for fixed (β) and random (σ^2) parameters are given with 95 % credible intervals (CrI). Fixed effects where CrI's do not overlap zero are highlighted in bold. Additionally, we report conditional coefficients of determination ($R^2_{GLMM(c)}$), as well as the total variance (r^2) in each aggression parameter explained by ES (BLUPs).

	No. calls	Time on decoy	Occurrence of attack	Approach distance	Enter nest box
**Fixed**	β (95% CrI)	β (95% CrI)	β (95% CrI)	β (95% CrI)	β (95% CrI)
Intercept	1.57 (0.93 ; 2.21)	0.82 (-1.23 ; 2.88)	-1.99 (-5.22 ; 0.83)	-0.95 (-1.25 ; -0.65)	-0.06 (-1.24 ; 1.31)
Clutch size	0.19 (-0.18 ; 0.60)	-	-	-	-
Age2	-	-2.06 (-4.60 ; 0.30)	-2.79 (-7.24 ; 0.62)	-	-1.84 (-3.48 ; 0.01)
ES (BLUPs)	**- 1.98 (-3.70 ; -0.34)**	**3.84 (0.52 ; 7.03)**	3.00 (-1.11 ; 9.26)	0.61 (-0.17 ; 1.37)	1.29 (-0.94 ; 3.48)
**Random**	σ^2 (95% CrI)	σ^2 (95% CrI)	σ^2 (95% CrI)	σ^2 (95% CrI)	σ^2 (95% CrI)
Female ID	3.30 (0.95 ; 6.12)	10.48 (2.53 ; 22.01)	17.26 (0.10 ; 52.61)	0.72 (0.17 ; 1.32)	2.27 (0.09 ; 6.84)
Residual	2.25 (0.99 ; 3.87)	7.08 (2.71 ; 13.03)	1.00 (1.00 ; 1.00)	0.68 (0.37 ; 1.05)	1.00 (1.00 ; 1.00)
$R^2_{GLMM(c)}$	0.97	0.96	0.93	0.57	0.17
r^2	0.10	0.13	0.01	0.04	0.04

Supplementary References

1. Hadfield, J. D. MCMC methods for multi-respoinse generalized linear mixed models: The MCMCglmm R package. *J. Stat. Softw.* **33,** 1–22 (2010).
2. Gelman, A. & Rubin, D. B. Inference from Iterative Simulation Using Multiple Sequences. *Stat. Sci.* **7,** 457–511 (1992).

3. Nakagawa, S. & Schielzeth, H. Repeatability for Gaussian and non-Gaussian data: A practical guide for biologists. *Biol. Rev.* **85,** 935–956 (2010).

4. Nakagawa, S. & Schielzeth, H. A general and simple method for obtaining R2 from generalized linear mixed-effects models. *Methods Ecol. Evol.* **4,** 133–142 (2013).

5. Nimon, K., Oswald, F. L. & Roberts, J. K. Interpreting regression effects. R package version 2.0-0. *https://CRAN.R-project.org/package=yhat* (2013).

6. Nakagawa, S. & Schielzeth, H. Repeatability for Gaussian and non-Gaussian data: A practical guide for biologists. *Biol. Rev.* **85,** 935–956 (2010).
